# Novel insights from human induced pluripotent stem cells on origins and roles of fibro/adipogenic progenitors as heterotopic ossification precursors

**DOI:** 10.3389/fcell.2024.1457344

**Published:** 2024-09-02

**Authors:** Chengzhu Zhao, Makoto Ikeya

**Affiliations:** ^1^ Laboratory of Skeletal Development and Regeneration, Key Laboratory of Clinical Laboratory Diagnostics (Ministry of Education), College of Laboratory Medicine, Chongqing Medical University, Chongqing, China; ^2^ Department of Clinical Application, Center for iPS Cell Research and Application (CiRA), Kyoto University, Kyoto, Japan

**Keywords:** fibro/adipogenic progenitors, induced pluripotent stem cells, muscle connective tissue, developmental origin, neural crest cells

## Abstract

Fibro/adipogenic progenitors (FAPs) that reside in muscle tissue are crucial for muscular homeostasis and regeneration as they secrete signaling molecules and components of the extracellular matrix. During injury or disease, FAPs differentiate into different cell types and significantly modulate muscular function. Recent advances in lineage tracing and single-cell transcriptomics have proven that FAPs are heterogeneous both in resting and post-injury or disease states. Their heterogeneity may be owing to the varied tissue microenvironments and their diverse developmental origins. Therefore, understanding FAPs’ developmental origins can help predict their characteristics and behaviors under different conditions. FAPs are thought to be the major cell populations in the muscle connective tissue (MCT). During embryogenesis, the MCT directs muscular development throughout the body and serves as a prepattern for muscular morphogenesis. The developmental origins of FAPs as stromal cells in the MCT were studied previously. In adult tissues, FAPs are important precursors for heterotopic ossification, especially in the context of the rare genetic disorder fibrodysplasia ossificans progressiva. A new developmental origin for FAPs have been suggested that differs from conventional developmental perspectives. In this review, we summarize the developmental origins and functions of FAPs as stromal cells of the MCT and present novel insights obtained by using patient-derived induced pluripotent stem cells and mouse models of heterotopic ossification. This review broadens the current understanding of FAPs and suggests potential avenues for further investigation.

## 1 Introduction

In 2010, fibro/adipogenesis-associated muscle-resident cells were identified in adult mice based on PDGFRα expression (Joe et al., 2010; Uezumi et al., 2010). These cells were isolated using the expression of specific surface antigens, such as Sca1 and Tie2, while excluding muscle satellite (SM/C-2.6^+^), hematopoietic (CD45^+^), and endothelial (CD31^+^) cell lineages and were termed fibro/adipogenic progenitors (FAPs). In 2014, FAPs from human skeletal muscle were also identified (Uezumi et al., 2014). FAPs reportedly orchestrate regenerative signals via paracrine functions to modulate the proliferation, differentiation, and myogenic capacity of muscle stem cells in various body regions (Farup et al., 2015; Wosczyna et al., 2019; Kim et al., 2022). They are the primary source of cells for regenerative matrix deposition and contribute to fibrous deformation/fatty infiltration in muscle tissues through differentiation into fibroblasts and adipocytes, particularly in the context of muscle damage and neuromuscular diseases (Cordani et al., 2014; Lemos et al., 2015; Contreras et al., 2016; Gonzalez et al., 2017; Malecova et al., 2018; Moratal et al., 2018; Moratal et al., 2019; Farup et al., 2021; Engquist et al., 2024; Flores-Opazo et al., 2024).

FAPs’ diversity and heterogeneity have hindered the understanding of their characteristics and predicting their functions and behaviors. FAPs have been found to exhibit altered gene regulatory networks or express state-specific markers under different conditions, which in turn regulates their fate and plasticity. Therefore, Resting FAPs in undamaged mouse muscles (Scott et al., 2019; De Micheli et al., 2020a; Oprescu et al., 2020) and FAPs activated during muscle regeneration (Malecova et al., 2018; Scott et al., 2019; De Micheli et al., 2020a; De Micheli et al., 2020b; Oprescu et al., 2020; Rubenstein et al., 2020) are heterogeneous populations with different functions or at different stages. Their heterogeneity may be caused by varied muscle tissue microenvironments and their diverse developmental pathways. Tracing the developmental origins of FAPs is an important entry point for understanding their intrinsic properties. During embryonic muscle development, FAPs are identified as the main sources of developmental extracellular matrix (ECM). Genetic lineage tracing has shown that the transcription factor odd-skipped-related 1 (Osr1) marks a subpopulation of FAP-like cells that supports myogenesis by promoting myogenic progenitor proliferation and survival (Vallecillo-García et al., 2017). Adult FAPs are reported to share a common lineage with embryonic muscle connective tissue (MCT) cells, which are responsible for the development of the macroscopic and microscopic attributes of muscle (Nassari et al., 2017; Sefton and Kardon, 2019).

MCT cells, the primary cell type in the muscle tissue, are responsible for producing and depositing ECM components, such as fibrous collagen and proteoglycans (Light and Champion, 1984). They are associated with the healing process following muscle injury and exhibit phenotypic characteristics akin to those of fibroblasts (Williams and Goldspink, 1984; Gatchalian et al., 1989; Schmitt-Gräff et al., 1994; Gabbiani, 1998; Chapman et al., 2017). With contributions from many researchers, it has been established that adult MCT is primarily derived from muscle-resident PDGFRα^+^ FAPs. These cells exhibit the characteristics of tissue-resident mesenchymal stem/progenitor cells (MSCs/MPCs) with multilineage differentiation potential and a fibroblast-like phenotype (Nesti et al., 2008; Jackson et al., 2009; Judson et al., 2013; Scott et al., 2019). However, these discoveries have led to some confusion in terminology. Contreras et al. (2021) proposed that these muscle-resident multipotent progenitor cells, whether called FAPs or fibroblasts, are essentially the same cell populations in studies related to MCT. Although careful identification of these cell types and the use of context-appropriate terminology are necessary, this review integrates previous literature on the developmental origins of FAPs by adopting the perspective that MCT cells serve as a collective term encompassing fibroblasts, MSCs/MPCs, and FAPs.

Relevant studies have provided valuable prospects into the origins of FAPs. As the primary precursor cells give rise to heterotopic ossification (HO) (Wosczyna et al., 2012), recent investigations utilizing patient-derived induced pluripotent stem cells (iPSCs) and mouse models have introduced new perspectives on FAPs’ embryonic origins. We discuss the conventional developmental perspectives on the origins and functions of MCT cells/FAPs during myogenesis and introduce novel insights emerging from diseased iPSCs and mouse models to deepen our understanding of related research field.

## 2 Embryonic origins and roles of MCT cells and FAPs during muscle development

**FIGURE 1:**
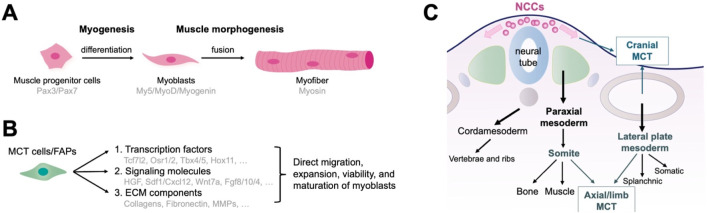
Function and embryonic origin of fibro/adipogenic progenitors (FAPs) as muscle connective tissue (MCT) cells during muscle development. **(A)** The development of muscle tissue. Myogenesis encompasses the formation of muscle progenitor cells, differentiation into myoblasts, and the fusion of myoblasts into multinucleated muscle fibers. **(B)** MCT cells control multiple aspects of muscle morphogenesis by directing myoblasts to migrate to the target region and promoting their expansion, viability, and maturation through the production of signaling molecules and ECM components. **(C)** Embryonic origin of MCTs representing the traditional developmental biology perspectives. Axial MCT originates from the somites and lateral plate mesoderm, limb MCT from the lateral plate mesoderm, and cranial MCT from the neural crest.

Muscle formation and development involve two parallel processes: myogenesis and morphogenesis. Myogenesis involves the formation of muscle progenitor cells that express Pax3 and Pax7, followed by differentiation into myoblasts. Committed myoblasts then proliferate, express myogenic regulatory factors (including Myf5, MyoD, and myogenin), undergo morphological changes, and fuse to form multinucleated muscle fibers (Rudnicki et al., 1992; Braun and Gautel, 2011; Abmayr and Pavlath, 2012; Sampath et al., 2018) (Figure 1A). Early development and initial differentiation into myoblasts are not controlled by MCT cells. Instead, the MCT stromal cells control multiple aspects of muscle morphogenesis (Sefton and Kardon, 2019). They direct myoblasts to migrate to the target region, as well as promote expansion, viability, and maturation of adjacent myoblasts into myofibers by secreting complex signaling molecules and ECM components (Bladt et al., 1995; Dietrich et al., 1999; Vasyutina et al., 2005; Hasson et al., 2010; Swartz et al., 2012; Iwata et al., 2013; Vallecillo-García et al., 2017; Stumm et al., 2018) (Figure 1B). MCT may act as a pre-model to determine the location of myofiber differentiation, which in turn determines the quantity, position, scale, and structure of a muscle. The following sections introduce the origins and functions of MCT cells/FAPs in major regions of the body.

### 2.1 Roles of MCT/FAPs in development of axial muscles

The axial or trunk muscles adjacent to the dorsal notochord, vertebrae, and abdomen originate from somites of the paraxial mesoderm (Buckingham, 2006; Bryson-Richardson and Currie, 2008; Wotton et al., 2015). Lineage studies using *Prx1Cre* transgenic mice identified two distinct developmental origins of axial muscle MCT: 1. MCT surrounding the distal intercostal, pectoralis, transverse, internal/external oblique, and rectus abdominis (medial/lateral muscles) originate from the lateral plate mesoderm; 2. MCT surrounding the intercostal and longissimus (proximal muscles) are derived from somites (Durland et al., 2008) (Figure 1C). Additionally, *ScxGFP*-transgenic mice, marking syndetome and tenocyte cells, also labeled MCT cells in some lateral muscles (Deries et al., 2010; Ono et al., 2023). Morphogenesis of the medial/lateral and proximal muscles is closely linked to ECM alterations concerning MCT of the lateral plate mesoderm and somite. MCT-derived fibronectin may contribute to this stage (Deries et al., 2010; Deries et al., 2012). *Fat1* is associated with planar cell polarity and plays a crucial role in the distribution and differentiation of myoblasts. *Fat1* absence in the lateral plate mesoderm of *Prx1*-expressing cells causes myofibril dysfunction and low count of myogenic cells, implying that mesenchymal cells derived from the lateral plate mesoderm control axial muscle morphogenesis by secreting ECM and regulating critical signaling pathways (Helmbacher, 2018). Furthermore, the axial muscles, tendons, and bones are derived from different somite regions, and their spatial interrelationships enable coordinated development. MCT derived from somite and lateral plate mesoderm likely regulate these interactions.

### 2.2 MCT/FAPs and limb muscle formation

The first step in limb muscle morphogenesis is the migration of myoblasts from the somites to the limbs. Once in the limbs, myoblasts undergo a complex morphogenetic process that ultimately forms the limb muscle pattern.

During the initial migration, attraction and repulsion signals in the limb bud mesoderm, produced by MCT cells derived from embryonic lateral plate mesoderm, are essential at this stage (Helmbacher and Stricker, 2020). For instance, stromal cell-derived factor 1 (SDF1 or Cxcl12) produced in this area, binds to CXCR4 produced by migrating myoblasts, actively regulating their survival and entry into the limbs (Vasyutina et al., 2005). Meanwhile, the ligand EphrinA5 expressed in the MCT repels myoblasts that express the receptor Epha4, preventing their settlement in the limb regions (Swartz et al., 2001).

In the subsequent step, the prepattern established by limb MCT determines the differentiation location and the number of myoblasts. Several key transcription factors, which have been identified in mouse MCT, importantly regulate this process. Knockout of the T-box transcription factors Tbx5 and Tbx4, respectively, cause misplacement of the forelimb and hindlimb muscles (Agarwal et al., 2003; Hasson et al., 2007; Naiche and Papaioannou, 2007). Tcf7l2 mutations cause truncation of the muscle near the knee and affect myofiber development and profile (Mathew et al., 2011). Tbx3-mutant mice lack two specific anterior muscles: the lateral triceps and brachialis (Colasanto et al., 2016). Compound mutations in Hoxa11 and Hoxd11 cause abnormalities in the forearm and calf muscles (Swinehart et al., 2013).

In summary, the MCT cells of the lateral plate mesodermal origin is essential for limb muscle morphogenesis. Activities of the signaling and transcription factors determine the position and number of myoblasts in the limbs, thereby influencing muscular pattern formation.

### 2.3 MCT/FAPs in cranial muscle development

The head muscles and MCT develop from diverse origins. The cranial mesoderm is the primary source of head muscles. It is positioned bilaterally along the neural tube and spans somites across the forebrain during embryonic development (Michailovici et al., 2015; Ziermann et al., 2018). MCT cells within the head muscles typically derives from the cranial neural crest. Moreover, neck muscles derive from somites and the head mesoderm. The MCT cells of the neck originates in the neural crest and lateral plate mesoderm (Matsuoka et al., 2005; Durland et al., 2008; Lescroart et al., 2015; Sefton and Kardon, 2019) (Figure 1C).

Formation of head muscles is initiated by the simultaneous migration of myogenic and neural crest cells (NCCs), accompanied by myoblast specialization. Within the pharynx, the myogenic cells constitute the central structure of each arch. Neural crest-derived MCT cells surround and invade them, causing significant mixing of both cell types. Simultaneously, myogenic cells begin myogenesis and specialize into myoblasts (Trainor et al., 1994; Grenier et al., 2009). Although neural crest-derived MCT cells are not required for the initiation of craniomandibular myogenesis (Heude et al., 2018), they critically regulate the migration, stereotyping, and differentiation of craniofacial myogenic precursors (Tzahor et al., 2003; Rinon et al., 2007). Deletion of *Pitx2* in NCCs in Wnt1Cre mice proves that it crucially regulates positioning of the extraocular muscles (Evans and Gage, 2005). Deletion of the transcription factors Dlx5 and Dlx6 in the murine cranial NCCs causes mandibular absence, indicating that they are critical for morphogenesis of jaw muscles (Heude et al., 2010). Lingual muscle development commences with the migration of myogenic and NCCs into the tongue bud (Han et al., 2012). Conditional knockout studies in mice indicated that cilium-dependent GLI in the neural crest is crucial for the viability and movement of myogenic cells toward the bud (Millington et al., 2017). Thus, signaling by the MCT cells originated from neural crest modulates myoblast expansion and maturation, eventually developing the cranial muscle patterns.

## 3 Novel insights into the origin of FAPs: perspectives from iPSCs and mouse models of HO

Studies based on traditional perspectives, which classify FAPs as MCT cells, have provided a comprehensive understanding of the embryonic origins of MCT/FAPs and their roles in muscle development (Table 1). However, it remains a challenge to confirm whether the FAPs present in different regions of adult muscle are completely originate from the MCTs described in the previous sections. In recent years, studies related to HO using patient-derived iPSCs and mouse models have provided new insights into the origin of FAPs.

**TABLE 1:** Embryonic origins and function of fibro/adipogenic progenitors as muscle connective tissue stromal cells in major regions of the body.

Body regions	Embryonic origins	Functional studies		
		Representative genes	Mutant/knockout effects	Reference
Axial Muscle	1. Lateral plate mesoderm 2. Somites	Fat1	Myofibril dysfunction and low count of myogenic cells	Helmbacher (2018)
		Scx	Disfunction of the back muscles	Deries et al. (2010)
Limb Muscle	Lateral plate mesoderm	CXCR4	Reduced number of muscle progenitors that colonize the dorsal limb	Vasyutina et al. (2005)
		Tbx4	Misplacement of the hindlimb muscles	Naiche and Papaioannou (2007)
		Tbx5	Failure of forelimb formation	Agarwal et al. (2003)
		Tcf7l2	Truncation of the muscle near the knee and affect myofiber development and profile	Mathew et al. (2011)
		Tbx3	Lack of the lateral triceps and brachialis	Colasanto et al. (2016)
		Hoxa11 and Hoxd11	Abnormalities in the forearm and calf muscles	Swinehart et al. (2013)
		Pitx2	Positioning of the extraocular muscles	Evans and Gage (2005)
Cranial Muscle	1. Neural crest 2. Lateral plate mesoderm	Dlx5 and Dlx6	Mandibular absence	Heude et al. (2010)
		GL1	Viability and movement of myogenic cells toward the bud	Millington et al. (2017)

### 3.1 FAPs are the principal precursor cells for HO

HO is abnormal bone formation in soft tissues, such as muscles, tendons, and ligaments. HO has two main subtypes: acquired and hereditary. Acquired HO is a common complication of major injuries to connective tissues, traumatic injuries to the central nervous system, and surgical interventions, and may cause pain and postoperative disability. Fibrodysplasia ossificans progressiva (FOP) is a rare but destructive form of hereditary HO that is caused by *ACVR1* mutations, which activate the bone morphogenetic protein (BMP) signaling pathway and subsequent ossification. FOP usually begins during childhood and manifests as soft-tissue swelling and inflammatory episodes that may progress to form ectopic bones (Shore et al., 2006; Kaplan et al., 2012).

FAPs residing in the interstitium of skeletal muscles, labeled with *Tie2Cre*, *PDGFRα*, and *Sca-1*, are identified as the major precursor cells driving HO (Uezumi et al., 2010; Wosczyna et al., 2012). Despite evidence from various studies suggesting that other cell types, including Prrx1^+^ skeletal progenitor cells (Chakkalakal et al., 2016; Hesse, 2016), Scx^+^ tendon-derived progenitor and muscle-resident interstitial Mx1^+^ population (Dey et al., 2016) and Tie2-expressing endothelial-related cells (Medici et al., 2010), as potential contributors to HO lesions, lineage-tracing studies have demonstrated the decisive role of FAPs in HO. PDGFRα-expressing cells labeled with fusion of CreERT and glutamate–aspartate transporter (GLAST–*CreERT*) or α-smooth muscle actin (αSMA–*CreERT2*) significantly contribute at all stages of BMP-induced HO (Kan et al., 2013; Matthews et al., 2016). The FAPs are identified as the major cell population in muscles capable of ectopically depositing cartilage or bone after BMP2 injection or overexpression (Lees-Shepard et al., 2018; Wang et al., 2020; Eisner et al., 2020). In models of HO induced by burns or tendon excisions, highly proliferative PDGFRα^+^ MSC-like cells accumulate at injury sites, subsequently generating HO (Agarwal et al., 2016; Agarwal et al., 2017). Furthermore, mouse model expressing ACVR1 (R206H) in FAPs recapitulates the full HO spectrum observed in patients with FOP, including injury-induced and spontaneous HO production (Lees-Shepard et al., 2018). These studies collectively demonstrate that FAPs give rise to HO, whether acquired or owing to FOP. Originally defined by their fibrogenic and adipogenic capabilities, the term ‘FAPs’ did not consider their chondrogenic and osteogenic potentials revealed in recent HO studies. A reconsideration of this terminology might better reflect the evolving understanding of these progenitors and their diverse functions.

### 3.2 New perspectives from iPSC-based disease models derived from patients with FOP

Studies of iPSCs derived from patients with FOP have provided new insights into FAPs’ origins. Many studies on the pathogenic mechanisms of FOP have relied on animal experiments; however, specific mechanisms in humans remain largely unknown. Additionally, obtaining clinical samples for FOP research is difficult, with triggering factors such as surgery possibly complicating the patients’ conditions. Human iPSCs derived from patients with FOP provide unique opportunities to study the mechanisms underlying the human disease. We previously generated a series of iPSC lines using the dermal fibroblasts of patients with FOP (FOP-iPSCs) (Matsumoto et al., 2013). Rescued iPSC clones (resFOP-iPSCs), with the pathogenic ACVR1^R206H^ mutation was corrected by BAC-based homologous recombination to correct the FOP mutation (617G > A) existing in exon 7, were generated using FOP-iPSCs as genetically matched controls. Using a stepwise induction method that mimics embryonic development (Fukuta et al., 2014), induced MSCs (iMSCs) were generated from resFOP/FOP-iPSCs using the NCC lineage. Using these methods, we previously constructed a human-derived *in vitro* disease model of FOP (Matsumoto et al., 2015). In FOP-iMSCs, BMP and transforming growth factor (TGF)-β signaling were stronger compared to resFOP-iMSCs. FOP-iMSCs exhibited enhanced chondrogenic capacity, which is crucial for promoting endochondral ossification. Furthermore, enhanced mineralization was confirmed in FOP-iMSCs cultured in a mineralization medium.

**FIGURE 2:**
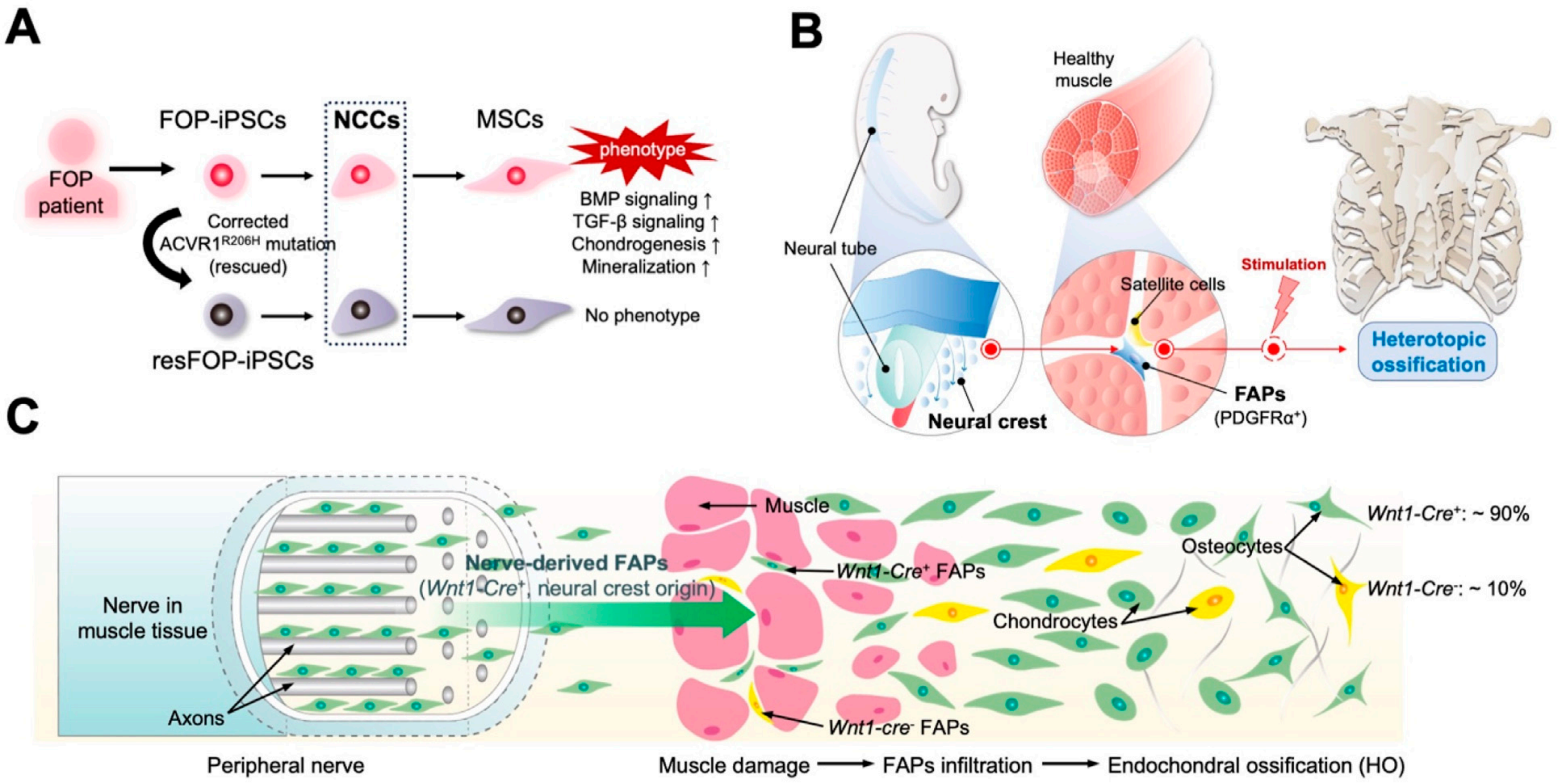
Novel perspectives developed by studying patient-derived induced pluripotent stem cells (iPSCs) and mouse models. **(A)** Mesenchymal stem cells (MSCs) derived from fibrodysplasia ossificans progressiva (FOP) patient-derived iPSCs (FOP-iPSCs) via neural crest cells (NCCs) are the fundamental origin cells of heterotopic ossification (HO) in FOP. Rescued iPSC clones (resFOP-iPSCs), with the pathogenic ACVR1^R206H^ mutation was corrected, were generated using FOP-iPSCs as genetically matched controls. Only induced MSCs (iMSCs) generated from FOP-iPSCs through NCC lineage exhibit the phenotype of FOP. **(B)** Critical role of FAPs derived from the neural crest in HO. **(C)** FAPs originate from neural crest lineage (*Wnt1-Cre* ^+^) located in the peripheral nerves contribute to HO.

Research has shown that chondroprogenitor cells derived from FOP-iPSCs via sclerotome, the developmental origin of tendons and ligaments, did not display enhanced chondrogenesis (Nakajima et al., 2018). These results indicate target cells induced via a specific pathway that exhibit a disease-prone phenotype might to some content reflect the cellular origins of the disease. MSCs derived from NCCs could be a potential cellular origin for ectopic bone in FOP (Figure 2A). Consist with these observations, by tracing cell lineages in mice, we investigated the correlation between NCC-derived FAPs and HO by different stimuli in mouse limbs. NCCs were marked in mice using *P0-Cre* or *Wnt1-Cre* (P0, migrating NCCs; Wnt1, dorsal neural tube before migration to neural crest) with a floxed *LacZ* reporter gene. In HO tissues induced by BMP7 injection, approximately 80% of cells positive for *P0-Cre* and *Wnt1-Cre* were co-labeled with the osteoblast marker SP7 and co-localized with regions positive for COL2 and COL1, which label the cartilage and bone matrix. Thus, NCCs are the origin for ectopic formation of cartilage and bone. Moreover, expression *FOP-ACVR1* specifically in the *P0*-lineage cells was sufficient to cause HO under CTX-induced muscle injury. BMP7-induced *P0-Cre*-positive and *Wnt1-Cre*-positive cells, as well as RFP-labeled *P0-Cre-FOP-ACVR1*-expressing cells, co-stained with approximately 90% of the PDGFRα- and Vimentin-expressing cells in HO tissues (Zhao et al., 2023). These findings affirm the pivotal contribution of FAPs originating from the neural crest to the pathogenesis of limb HO, regardless of etiology (Figure 2B). However, both the aforementioned results and other studies suggest that NCC-derived FAPs are not the exclusive source of HO. For instance, research has indicated that *Prx1-Cre* labeled mesoderm-derived adipose precursor cells are found in the muscle interstitium (Krueger et al., 2014). This may suggest the presence of HO-related mesenchymal precursors with distinct developmental origins. Additionally, another study using *WNT1-Cre/R26-YFP* mice did not find NCC-derived FAPs in the interstitial space of uninjured limb muscle (tibialis anterior) (Lemos et al., 2012). This discrepancy with the aforementioned study may be due to differences in the YFP and LacZ marking methods, or variations in the muscle microenvironment of the model mice under static conditions. Further validation from different perspectives, such as comparing the proportions of corresponding cells in injured limb muscle or HO tissue between the two model mice, would provide greater clarity on these findings.

### 3.3 FAPs derived from neural crest in the peripheral nerves contribute to HO

The neural crest is a stem cell/progenitor cell population that contributes to various conditions, including formation of the craniofacial cartilages and bones, smooth muscles, peripheral and intestinal neurons, and neuroglia (Le Douarin and Kalcheim, 1999). Cells derived from the neural crest are localized in various adult tissues and possess differentiate potential, associated with the new tissue formation following stimuli, injuries, or stresses (Neirinckx et al., 2013; Parfejevs et al., 2018). Studies based on muscle development have shown that neural crest-derived MCT cells/FAPs are primarily found in the craniofacial muscles, rather than in the axial or limb muscles. However, research discussed earlier has indicated that in the limb muscles, FAPs originating from neural crests play a significant role in HO. Consistently, studies of mouse models have revealed osteogenic precursor cells, which have originated from neural crests, within the peripheral nerves (Lazard et al., 2015; Olmsted-Davis et al., 2017; Carr et al., 2019).

The connective tissue sheath that encases each peripheral nerve bundle, the perineurium, contains MSCs that are tightly intertwined with axons and Schwann cells. These cells originate from the neural crest, and this unique embryonic origin may enhance their regenerative capacity (Davis et al., 2018; Joseph et al., 2004; Richard et al., 2014). These cells exhibit features of FAPs and are associated with tissue regeneration and ectopic bone formation. Storer et al. demonstrate that Pdgfr*a*-expressing mesenchymal cells in uninjured digits establish the regenerative blastema and are essential for regeneration (Storer et al., 2020). Interestingly, single-cell RNA sequencing and lineage tracing of injured sciatic nerves in adult mice transfected with an EGFP reporter knocked into the *Pdgfra* gene revealed that a substantial population of *Pdgfra*-*EGFP*-positive cells exhibit a mesenchymal precursor and embryonic mesenchyme transcriptional signature. These cells proliferate after injury, express genes related to chondrogenesis and osteogenesis, such as *Sox9* and *Alpl*, and can differentiate into osteo/chondro/adipogenic lineages. Crossing *Wnt1CreR26-LSL-tdTomato* (TdT) mice with the above mice showed that most *Pdgfra*-*EGFP*-positive endoneurial FAPs were *Wnt1Cre*-*TdT*-positive, indicating a neural crest origin in the injured nerves. These FAPs contribute to the formation of the regenerative blastema and, ultimately, to the regenerated bone following digit-tip amputation. Similar results were obtained in mice with another neural crest lineage tracing line, *DhhCre* mice (Carr et al., 2019). These findings support the hypothesis that the peripheral nerves facilitate tissue repair and regeneration by providing FAPs derived from the neural crest.

In the HO mouse model induced by muscle injection of Ad5BMP-2, cells expressing the osteoblast-specific transcription factor osterix appeared in the neurons at the injection site (Lazard et al., 2015). Subsequently, these cells migrated away from the endoneurial compartment and entered the site of new bone formation. These cells expressed both PDGFRα and the NCC marker low-affinity nerve growth factor receptor p75, suggesting that this HO precursor cell may be FAPs of NCC origin. Lineage tracing in mice that contained a tamoxifen-regulated *Wnt1-Cre* recombinase crossed with a *TdT* reporter (*Wnt1* ^*CreErt*^ *:Ai9Tm*) confirmed that following BMP-2 intramuscular injection, *TdT*-positive cells within the endoneurium co-expressed SP7 (osterix), pre-chondrocytes (Sox9), and transient brown fat (tBAT, UCP1), which is closely associated with subsequent HO (Olmsted-Davis et al., 2017). Additionally, human nerves near the HO site contain many phosphoSMAD1/5/8-positive cells. Constitutively activated ACVR1 in the cranial NCCs of mice induces ectopic cartilage formation in the craniofacial region by enhancing BMP signaling (Mishina and Snider, 2014). This finding may explain why patients with FOP develop HO in the craniofacial regions specifically through endochondral ossification. These studies indicate that, in both mouse and human acquired HO and FOP, the precursor cells for HO originate from NCCs in peripheral nerves (Figure 2C).

NCCs may persist in the endoneurium because of their immune-privileged position behind the blood–nerve barrier. Meanwhile, PDGFRα, a factor crucial for glia–endothelium interactions and the maintenance of the blood–brain barrier, is expressed in FAPs of neural crest origin and may play a key role in their transition from a neural to mesodermal fate (Lazard et al., 2015). Wnt1 is a major inducer of the neural crest (García-Castro et al., 2002; Ikeya et al., 1997). When not inhibited, Wnt1 signaling causes the formation of sensory neurons from neural stem cells (Lee et al., 2004). However, when inhibited by BMP2, Wnt1 signaling in NCCs leads to the formation of other cell types including osteogenic precursors (Kléber et al., 2005). Therefore, in addition to the FAPs present in the muscle interstitium, FAPs in adult muscle tissues may originate from NCCs deposited in the endoneurium during neural crest migration and sensory nerve formation, which re-enact embryonic processes for tissue regeneration upon activation by specific signals. This may explain the NCC-derived FAPs in the peripheral nerves that contribute to HO and their mechanisms of action.

### 3.4 Application of iPSCs-derived FAPs as a tool to investigate HO

iPSCs derived from patients with HO offers potential for understanding of the disease. Utilizing MSCs derived from FOP-iPSCs, a mechanism has been discovered in which FOP-ACVR1 abnormally transduces BMP signaling in response to Activin-A. This mechanism is distinct from the previously identified FOP-ACVR1-mediated ligand-independent constitutive activity and BMP ligand-dependent hyperactivity in BMP signaling. Activin-A enhanced the chondrogenesis of FOP-iMSCs via aberrant activation of BMP signaling *in vitro*, and induced endochondral ossification of FOP-iMSCs *in vivo* (Hatsell et al., 2015; Hino et al., 2015). Further research revealed that mTOR signaling as a critical pathway for the aberrant chondrogenesis induced by Activin-A in FOP-MSCs (Hino et al., 2017). Furthermore, drug screening based on the FOP-iMSCs has identified specific small-molecule compounds, such as rapamycin (Hino et al., 2017) and AZD0530 (Hino et al., 2018), as potential candidates which have entered clinical trials for treating FOP, supporting the reliability of the iPSC-based disease models.

However, FAPs and MSCs originated from iPSCs may not be entirely congruent cell populations. FACS sorting was used to isolate different subsets of MSC-like cells derived from FOP-iPSCs via somite (Nakajima et al., 2018). The results showed that PDGFRα^+^/CD31^−^ cells exhibited enhanced chondrogenesis compared to PDGFRα^−^/CD31^-^ cells, suggesting that specific differentiation protocols and isolation based on surface markers such as PDGFRα can be employed to obtain FAPs from iPSCs.

Building on the insights into the distinct origins of FAPs, iPSC-derived FAPs can serve as a valuable tool to investigate the role of embryonic origin on the behavior of FAPs in heterotopic ossification. By comparing the characteristics and functional capacities of FAPs derived from different embryonic origins, researchers can elucidate how these populations contribute to the pathogenesis of ectopic bone formation and potentially identify novel therapeutic targets. Furthermore, recent studies have shown that iMSC-mediated delivery of therapeutic agents, such as the ACVR2B-Fc fusion protein, can effectively alleviate HO symptoms in FOP model mice (Gao et al., 2024). Based on the different embryonic origins of FAPs, these cells may also provide a promising avenue for developing targeted therapies aimed at mitigating HO.

## 4 Conclusion

FAPs are essential for muscle homeostasis and regeneration in adults. They are vital for the regulation of muscle development. The previous literatures, by adopting the perspective that FAPs are MCT cells, mapped their origin and fate during embryonic development and elucidated their roles in regulating muscle morphogenesis. The axial MCT is thought to originate from the somites and lateral plate mesoderm; the limb MCT originates from the lateral plate mesoderm; and the cranial MCT originates from the neural crest and lateral plate mesoderm. Recent studies using iPSCs derived from patients with FOP and HO mouse models revealed the contribution of NCC-derived FAPs to HO. This novel finding, departing from traditional developmental perspectives, suggests that the heterogeneity of FAPs may be attributed to specific environments and signals that activate FAPs of different embryonic origins, leading to diverse responses. These perspectives aid in understanding the intricate roles of FAPs in muscle homeostasis, regeneration, and pathological conditions.

## Abbreviations

BMP, Bone morphogenetic protein; ECM, Extracellular matrix; FAPs, Fibro/adipogenic progenitors; FOP, fibrodysplasia ossificans progressiva; HO, Heterotopic ossification; iPSCs, Induced pluripotent stem cells; MCT, Muscle connective tissue; MPC, Mesenchymal progenitor cells; MSC, Mesenchymal stem cells; NCCs, Neural crest cells; TGF, transforming growth factor.
